# Partial volume correction for Lu-177-PSMA SPECT

**DOI:** 10.1186/s40658-024-00697-1

**Published:** 2024-11-12

**Authors:** Yibin Liu, Zhonglin Lu, Gefei Chen, Kuangyu Shi, Greta S. P. Mok

**Affiliations:** 1 Department of Electrical and Computer Engineering, Biomedical Imaging Laboratory (BIG), Faculty of Science and Technology, University of Macau, Avenida da Universidade, Taipa, Macau SAR China; 2 Department of Nuclear Medicine, Bern University Hospital, Inselspital, University of Bern, Freiburgstr. 18, Bern, 3010 Switzerland; 3 Ministry of Education Frontiers Science Center for Precision Oncology, Faculty of Health Science, University of Macau, Taipa, Macau SAR China

**Keywords:** ^177^Lu-PSMA-617, SPECT/CT, Partial volume correction, Recovery coefficient, Iterative Yang, Reblurred Van-Cittert

## Abstract

**Background:**

The limited spatial resolution in SPECT images leads to partial volume effect (PVE), degrading the subsequent dosimetric accuracy. We aim to quantitatively evaluate PVE and partial volume corrections (PVC), i.e., recovery coefficient (RC)-PVC (RC-PVC), reblurred Van-Cittert (RVC) and iterative Yang (IY), in ^177^Lu-PSMA-617 SPECT images.

**Methods:**

We employed a geometrical cylindrical phantom containing five spheres (diameters ranging from 20 to 40 mm) and 40 XCAT phantoms with various anatomical variations and activity distributions. SIMIND Monte Carlo code was used to generate realistic noisy projections. In the clinical study, sequential quantitative SPECT/CT imaging at 4 time-points post ^177^Lu-PSMA-617 injections were analyzed for 10 patients. Iterative statistical reconstruction methods were used for reconstruction with attenuation, scatter and geometrical collimator detector response corrections, followed by post-filters. The RC-curves were fit based on the geometrical phantom study and applied for XCAT phantom and clinical study in RC-PVC. Matched and 0.5-2.0 voxels (2.54–10.16 mm) mismatched sphere masks were deployed in IY. The coefficient of variation (CoV) was measured on a uniform background on the geometrical phantom. RCs of spheres and mean absolute activity error (MAE) of kidneys and tumors were evaluated in simulation data, while the activity difference was evaluated in clinical data before and after PVC.

**Results:**

In the simulation study, the spheres experienced significant PVE, i.e., 0.26 RC and 0.70 RC for the 20 mm and 40 mm spheres, respectively. RVC and IY improved the RC of the 20 mm sphere to 0.37 and 0.75 and RC of the 40 mm sphere to 0.96 and 1.04. Mismatch in mask increased the activity error for all spheres in IY. RVC increased noise and caused Gibbs ringing artifacts. For XCAT phantoms, both RVC and IY performed comparably and were superior to RC-PVC in reducing the MAE of the kidneys. However, IY and RC-PVC outperformed RVC for tumors. The XCAT phantom study and clinical study showed a similar trend in the kidney and tumor activity differences between non-PVC and PVC.

**Conclusions:**

PVE greatly impacts activity quantification, especially for small objects. All PVC methods improve the quantification accuracy in ^177^Lu-PSMA SPECT.

**Supplementary Information:**

The online version contains supplementary material available at 10.1186/s40658-024-00697-1.

## Introduction

The prevalence of prostate cancer is increasing and it is currently the fifth leading cause of cancer-related mortality in men [1]. Metastatic castration-resistant prostate cancer (mCRPC) is the advanced stage of disease in which cancer cells become resistant to hormone therapy, with substantial metastatic lesions observed throughout the body, especially in bone [2]. Prostate-specific membrane antigen (PSMA), a membrane-bound glycoprotein, is overexpressed in prostate cancers, making it a promising target for prostate cancer theranostics [3]. Recently, radioligand ^177^Lu-PSMA-617 is FDA approved for treating mCRPC by delivering high energy β^-^ particles to kill PSMA expressed cancer cells. Quantitative SPECT imaging provides 3D ^177^Lu-PSMA-617 distribution in vivo, enabling peri-therapeutic absorbed dose calculation to ensure the efficacy of individualized therapy and toxicity prevention [4]. Recent studies have shown strong negative correlations between tumor absorbed dose and prostate-specific antigen (PSA) level [5]. Thus, tumor dosimetry is important for predicting the treatment outcome.

Limited spatial resolution significantly affects the activity quantitation of ^177^Lu-PSMA-617 SPECT. Spatial resolution can be defined by the point spread function (PSF) of the reconstructed image which varies over the field-of-view (FOV). The full-width-at-half-maximum (FWHM) of the PSF decreases with distance away from iso-center in the reconstructed image [6]. Partial volume effect (PVE) is caused by the limited spatial resolution of scanners and the finite size of image voxels. PVE leads to the over- or underestimation of activity, especially on SPECT images [7]. The absorbed dose can be underestimated > 60% for a large tumor (10.2 mL) and 99% for a small tumor (0.06 mL) in ^177^Lu SPECT images [8].

Partial volume corrections (PVC) can be applied either during or post reconstruction. Studies have shown that applying resolution modeling and penalized likelihood using anatomical priors during reconstruction can reduce PVE [9]. On the other hand, several post-reconstruction-based PVC methods have been proposed, i.e., recovery coefficient (RC)-based PVC (RC-PVC), iterative deconvolution-based reblurred Van-Cittert (RVC) and anatomical-based iterative Yang (IY). The RC is defined as the ratio of the estimated activity to the true activity of an object. RC-PVC involves dividing the estimated activity of selected volume-of-interest (VOIs) by the size-dependent RC, where corrected images cannot be obtained [10]. The iterative-deconvolution and anatomical-based methods are voxel-based corrections and can generate corrected images. RVC deconvolves the post-reconstruction images by the PSF of the scanner’s resolution. However, RVC leads to noise amplification [10]. On the other hand, IY leverages the count distribution with additional anatomical masks, while system PSF still needs to be input. Although previous studies show that the IY method is robust against noise [10], accurate aligned anatomical information is crucial. The discrepancy in the mask, arising from misregistration or segmentation errors, could result in an error in IY-corrected images [11]. The objective of this study is to conduct a systematic analysis of PVE and evaluate the effectiveness of RC-PVC, RVC and IY methods on post-reconstructed ^177^Lu-PSMA-617 SPECT images based on simulation and clinical data.

## Materials and methods

### Simulation study

#### Phantom population

**Fig. 1:**
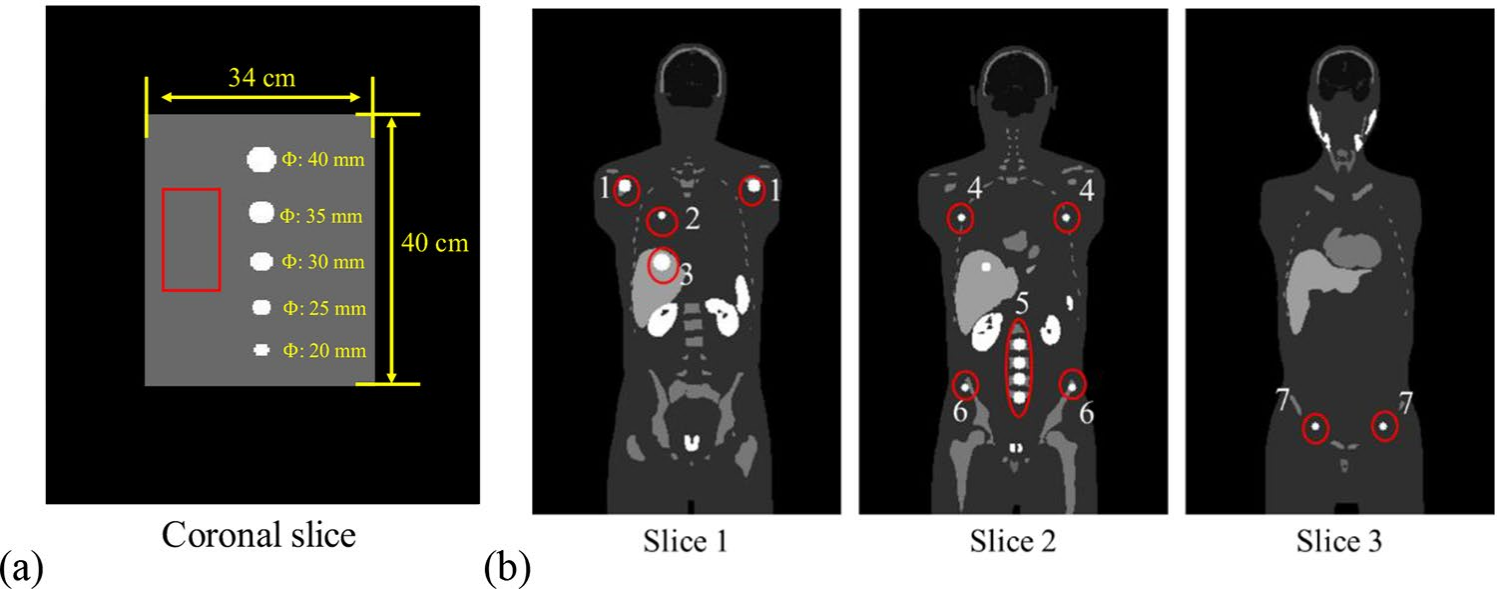
(**a**)The coronal slice of geometrical phantom. The 2D rectangular background region (60 × 30 pixels) marked by the red box was delineated to calculate the coefficient of variation (CoV). (**b**) Three sample coronal slices of the activity distribution for one selected XCAT phantom at the first imaging time point. The 14 spherical tumors at (1) shoulder bone marrow (×2), (2) lung, (3) liver, (4) rib (×2), (5) spine (×4), (6) basin bone marrow (×2) and (7) inguinal lymph nodes (×2) were indicated within red circles.

To investigate the impact of PVE for different object sizes on ^177^Lu SPECT, a digital geometrical cylindrical phantom with a diameter of 34 cm and a length of 40 cm was employed. This phantom contained five spheres with diameters of 20, 25, 30, 35, and 40 mm, positioned at the center of FOV along the axis-of-rotation (Fig. 1a). The phantom had a total activity of 3.5 GBq and sphere-to-background ratio of 10:1. The distance between the edges of each sphere was 4.5 cm. The matrix size and voxel size of the geometrical phantom were 256 × 256 × 180 and 2.54 × 2.54 × 2.54 mm^3^, respectively.

We also employed a population of 10 digital XCAT phantoms to simulate patients undergoing sequential quantitative SPECT/CT scans at four imaging time points (2, 20, 40, and 60 (*n* = 5)/200 (*n* = 5) h) following an injection of 7.4 GBq ^177^Lu-PSMA-617 [12]. Each phantom contained 14 spherical tumors with locations based on the metastatic patterns of mCRPC (Fig. 1b) [2]. These phantoms were designed to encompass various anatomical variations, including differences in body and organ sizes (Table 1) and activity distributions of different critical organs including the prostate, kidneys, salivary glands, spleen, bone marrow, liver, heart, and tumors (tumor-to-background ratios from 10:1 to 30:1) at four imaging time points based on the clinical data, resulting in a total of 40 phantoms (10 anatomies×4 imaging time points). The range of tumor volumes was from 0.5 to 65.5 ml. The lower bound is based on the fact that a tumor with a volume of > 0.5 mL (1 cm diameter) is categorized as an index tumor which is clinically significant and needs treatment [13]. The XCAT phantoms were generated with a matrix size of 256 × 256 × 440 and a voxel size of 2.54 × 2.54 × 2.54 mm^3^.

The attenuation maps at 208 keV were generated for the geometrical phantom (assuming water medium) and XCAT phantom to model attenuation in projection simulation and then used for attenuation correction (AC) in reconstruction.

**Table 1:** Summarized characteristics of the 10 XCAT phantoms.

Organ	Volume (mL) Mean ± SD (range)	Activity (MBq) Mean ± SD (range)
Prostate	17.3 ± 0.6 (16.5–18.1)	65.9 ± 5.3 (58.5–73.6)
Kidneys	160.5 ± 5.8 (151.2-168.1)	18.1 ± 4.1 (13.5–23.4)
Heart	747.1 ± 32.5 (711.2-790.9)	9.0 ± 0.6 (8.2–9.8)
Spleen	167.7 ± 5.9 (158.3-175.9)	9.2 ± 0.4 (8.9–9.9)
Bone marrow	1968.5 ± 266.6 (1585.3-2545.2)	4.6 ± 0.5 (4.0-5.1)

Total body weight (kg) Mean ± SD (range): 64.1 ± 19.1 (52.5–84.3)

Total body height (cm) Mean ± SD (range): 174.6 ± 8.8 (161.6-192.7)

#### Monte Carlo simulation

**Fig. 2:**
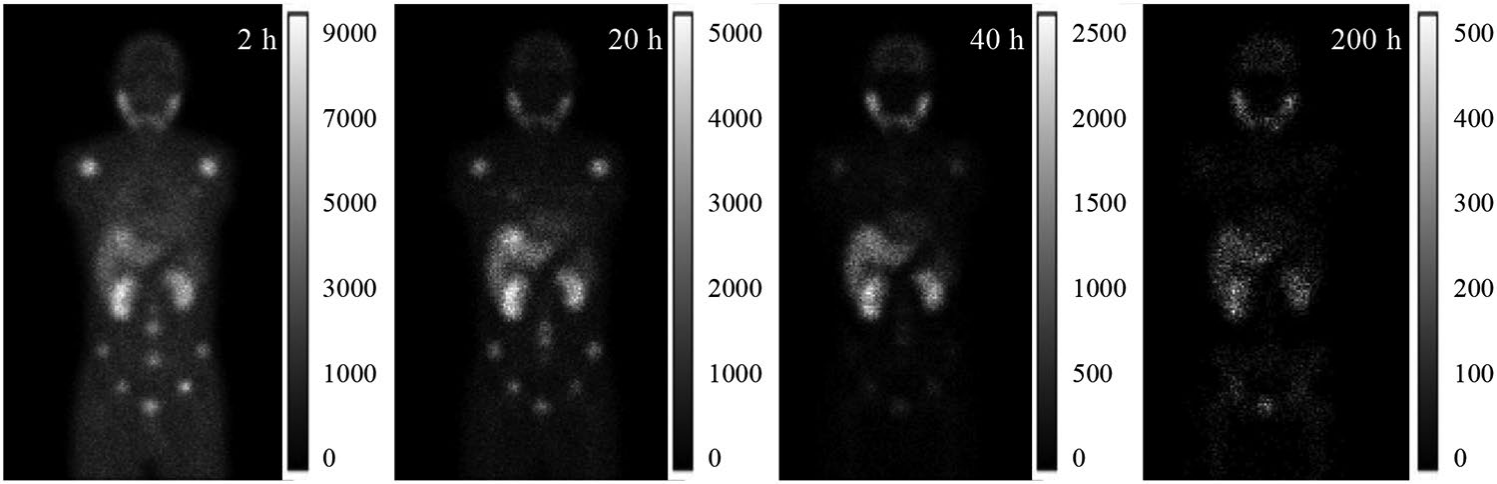
Sample noisy projections for one XCAT phantom at four imaging time points after ^177^Lu PSMA injection.

The SIMIND Monte Carlo program v7.0.3 [14] was used to simulate a standard clinical SPECT/CT system (Symbia Intevo 16, Siemens Healthineers, Germany) with medium energy general purpose (MEGP) parallel hole collimators for ^177^Lu imaging. Sixty noise-free projections were generated over 360° at one bed position for the geometrical phantom and three bed positions for XCAT phantoms, modelling attenuation, scatter, interactions within collimator-detector and back-scatter. The detector orbit was set to be circular with a radius-of-rotation (ROR) of 24 cm. For the XCAT phantom, projections from three bed positions were concatenated to generate total body projections (Fig. 2). The primary energy window was centered at 208 keV with a width of 20% (185.6-226.9 keV). Additionally, two scatter windows were set at 165.0-185.6 keV and 226.9-247.5 keV, respectively. Poisson noise was applied to the noise-free projections with total count number scaled to clinical projection counts (2 h: 1.4 × 10^8^, 20 h: 7.0 × 10^7^, 40 h: 5.5 × 10^7^, 60 h: 3.5 × 10^7^, and 200 h: 5.5 × 10^6^) to generate realistic noisy projections for the XCAT phantom. The same five noise levels were modeled for the geometrical phantom. To model the effect of continuous-to-discrete activity sampling of the activity distribution as seen in clinical data acquisition [15, 16], the matrix size for projections was collapsed from 256 × 180 (bin size 2.54 × 2.54 mm^2^) to 128 × 90 (bin size 5.08 × 5.08 mm^2^).

The ordered subset expectation maximization (OS-EM) algorithm (10 iterations and 4 subsets) was used for reconstruction, along with the triple energy window scatter correction (TEW-SC), AC and geometrical collimator detector response (GCDR) correction. A post-reconstruction Gaussian filter (20.8 mm FWHM for 200 h time point and 16 mm FWHM for other time points) was applied [17]. The reconstruction matrix size and voxel size of SPECT images were 128 × 128 × 210, and 5.08 × 5.08 × 5.08 mm^3^, respectively.

### Clinical study

Sequential quantitative SPECT/CT images acquired at 2, ~ 20, ~40, and ~ 60 (*n* = 5)/~200 (*n* = 5) h post 6.7–8.4 GBq ^177^Lu-PSMA-617 injection for ten patients were analyzed retrospectively (Table 2) under waiver of ethics from Cantonal Ethics committee Bern (Nr. Req-2023-01313). Sixty SPECT projections were obtained with body contour orbit for three bed positions over 360° using a conventional NaI SPECT system (Symbia Intevo 16, Siemens Healthiness, Germany) with MEGP collimators. The energy window settings were consistent with the simulation study. The acquisition time was 8 s/view at 1st − 3rd imaging time points and 15 s/view at 4th imaging time point covering from lower head to thighs. Corresponding sequential CTs (110–130 kV, 27–128 mA, pitch: 1.4) were acquired covering the same region as AC maps, with a voxel size of 1.27 × 1.27 × 2 mm^3^ (512 × 512×varying length). Projections were reconstructed using the system built-in xSPECT software based on the Maximum Likelihood Conjugate Gradient Minimizer algorithm with resolution recovery, TEW-SC and AC. The number of iterations (12, 24 or 60) with one subset and 3D-Gaussian post-filter with FWHM of 16–20.8 mm were the default settings from xSPECT Quant™ [17]. The matrix size and voxel size of the SPECT reconstruction were 128 × 128×varing length and 5.08 × 5.08 × 5.08 mm^3^. The SPECT/CT images were registered using the rigid plus B-spline transformation under the open-source program “Elastix” [18]. A reference ^177^Lu point source with an activity of 21.48 MBq was placed beside each patient to determine the calibration factor to convert SPECT counts to activity concentration (MBq/ml).

**Table 2:** Demographic information of 10 ^177^Lu-PSMA-617 patient datasets.

Characteristics	Minimum	Maximum	Mean ± SD
Age (years)	56	75	68 ± 6.9
Height (cm)	168	183	173 ± 6.5
Kidney volume (ml)	169.31	263.77	205.38 ± 33.58
Tumor volume (ml)	17.96	70.49	34.08 ± 14.58

### Partial volume correction

The RC was defined as follows:

1 $$\:RC=\frac{{A}_{SPECT}}{{A}_{true}}$$

Where $\:{A}_{SPECT}$ is the estimated activity in the VOI from the non-PVC and PVC results and $\:{A}_{true}$ is the true activity in the VOI.

The RC-curve was generated by fitting the RC for different spheres with sphere-to-background ratio of 10:1 from the previous geometrical phantom study (supplementary Fig. S1) using $\:{RC}_{VOI}=1-\frac{1}{a{e}^{bx}}$, where *a* and *b* are the two fit parameters and *x* is the sphere volume (ml). The RC-PVC was implemented as follows:

2 $$\:{A}_{RC-PVC}=\frac{{A}_{SPECT}}{{RC}_{VOI}}$$

$\:{A}_{RC-PVC}$ is the activity of VOI after RC-PVC.

The RVC method was implemented as follows:

3 $$\:{f}_{j}^{k+1}={f}_{j}^{k}+\alpha\:\cdot\:h\otimes\left({f}_{j}^{0}-h\otimes{f}_{j}^{k}\right)$$

where $\:{f}_{j}^{k}$ represents *k*^th^ (k ≥ 0) iteration image whereas $\:{f}_{j}^{0}\:$is the input reconstructed image, $\otimes$ is the 3D convolution operator, $\:\alpha\:$ affects the convergence rate and is empirically set to 1 [19], *j* is the voxel index and $\:h$ is the 3D PSF. The total counts were preserved by scaling the total counts of $\:{f}_{j}^{k}$ to $\:{f}_{j}^{0}$.

The algorithm will be terminated when:

4 $$\:\frac{\sqrt{\sum\:{\left({f}_{j}^{k+1}-{f}_{j}^{k}\right)}^{2}}}{\sqrt{\sum\:{{(f}_{j}^{0})}^{2}}}<0.01$$

The IY method was implemented as follows:

5 $$\:{f}_{j}^{k+1}={f}_{j}^{k}\left[\frac{{s}_{j}^{k}}{h\otimes{s}_{j}^{k}}\right]$$

6 $$\:{s}_{j}^{k}=\sum\:_{i=1}^{i=n}\left[{T}_{k,i}{p}_{i}\right]$$

7 $$\:{T}_{k,i}=\frac{1}{{v}_{i}}\left[\sum\:_{j\in\:{p}_{i}}{f}_{j}^{k}\right]$$

where *i* is the number of masks, $\:{p}_{i}$ is the *i*^*th*^ binary mask, $\:{v}_{i}$ is the number of voxels in the mask, $\:{T}_{k,i}$ is the mean value of the mask for *k*^th^ iteration and $\:{s}_{j}^{k}$ is the sum of masks with the value of $\:{T}_{k,i}$ in each $\:{p}_{i}$. The maximum *k* value is 10 in IY as suggested by Thomas et al. [19]

The regional counts were preserved after each iteration as follows:

8 $$\:{{f}_{cp}}_{j\in\:{m}_{j}}^{k+1}={f}_{j\in\:{m}_{j}}^{k+1}\left[\frac{\sum\:_{j\in\:{m}_{j}}{f}_{j\in\:{m}_{j}}^{k}}{\sum\:_{j\in\:{m}_{j}}{f}_{j\in\:{m}_{j}}^{k+1}}\right]$$

9 $$\:{m}_{j}=h\otimes{s}_{j}^{0}$$

where $\:{m}_{j}$ is the count preservation region in each iteration and $\:{f\_cp}_{j\in\:{m}_{j}}^{k}$ represents the $\:{f}_{j\in\:{m}_{j}}^{k}$ with count preservation. The effect of mismatched masks on IY method was evaluated. The mismatched masks were generated by shifting the matched mask by 0.5, 1.0, 1.5 and 2.0 voxel (2.54–10.16 mm) along the radial direction based on clinical references [20–23].

### Point spread function model

For both RVC and IY, the PSF was obtained by simulating an analytical infinitesimal ^177^Lu point source at the center of FOV using acquisition, reconstruction and post-filtering parameters consistent with the phantom and clinical study. For the clinical body contour orbit, the PSFs were obtained by simulating a ROR based on the mean imaging distance among 60 projections.

### Data analysis

For the geometrical phantom simulation, the CoV was measured on a uniform rectangular background region marked by the red box to indicate the noise level on non-PVC and PVC results (Fig. 1a):

10 $$\:CoV=\:\frac{\sigma\:}{\mu\:}\:$$

where σ is the standard deviation and $\:\mu\:$ is the mean of the 2D region-of-interest.

The RCs of spheres were evaluated with the non-PVC and PVC images for the geometrical phantoms. The mean-absolute-error (MAE) of tumor and kidney activity in the XCAT phantoms was defined as follows:

11 $$\:MAE=\:\frac{\sum\:_{1}^{n}\left|Activity\:error\%\right|}{n}$$

12 $$\:Activity\:error\%=\left(1-\frac{{A}_{SPECT}}{{A}_{true}}\right)\times\:100\%$$

n is 80 for kidneys (2 × 10 phantoms×4-time point) and 560 for tumors (14 × 10 patients×4-time point). The phantoms with a voxel size of 2.54 × 2.54 × 2.54 mm³ were down sampled to 5.08 × 5.08 × 5.08 mm³ by skipping every other row and column. The VOI masks of kidneys and tumors were directly obtained from down sampled phantoms without mismatch.

For the clinical data, as the gold standard for the activity was absent, we evaluated the mean activity difference between non-PVC and PVC results:

13 $$\:Mean\:activity\:difference\%\:=\frac{1}{n}\sum\:_{1}^{n}\left(\frac{{A}_{PVC}}{{A}_{non-PVC}}-1\right)\times\:100\%$$

where $\:{A}_{PVC}$ and $\:{A}_{non-PVC}$ are the activities from the PVC and non-PVC results respectively, and n is 80 for kidneys (2 × 10 patients×4-time points) and 40 for tumors (1 × 10 patients×4-time point) respectively. The kidney and tumor VOI masks were segmented manually on CTs. Sample SPECT and CT images, demonstrating a visible tumor on CT, are shown in supplementary Fig. S2.

## Results

### Geometrical phantom

**Fig. 3:**
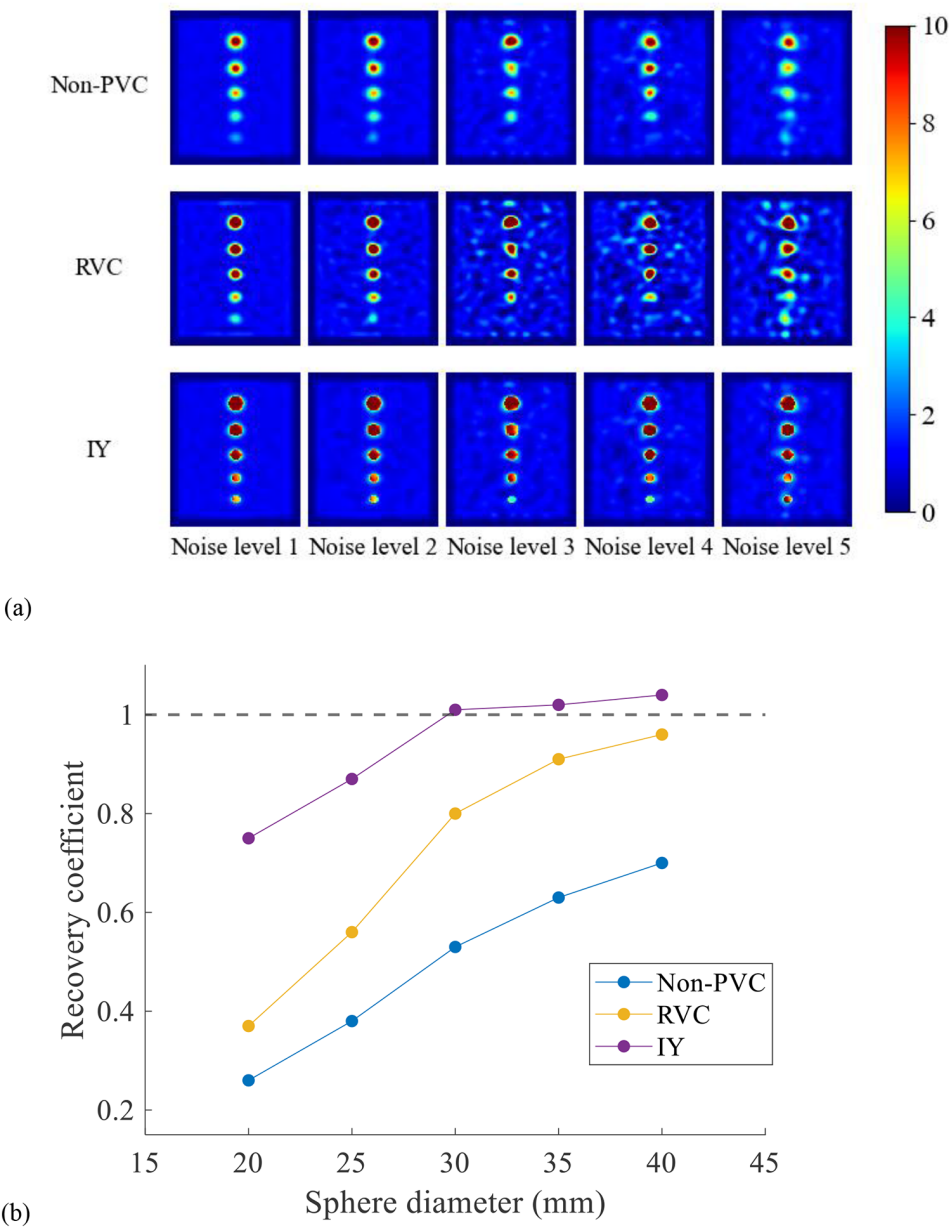
(**a**) The non-PVC and PVC results of the geometrical phantom. (**b**) The RC for each sphere on (**a**) non-PVC, RVC and IY images at noise level 1.

Sample non-PVC, RVC, IY images and corresponding RCs for different spheres at noise level 1 are shown in Fig. 3. Both PVC methods improved the estimated activity in the hot spheres. Without PVC, the RCs ranged from 0.26 (20 mm sphere) to 0.70 (40 mm sphere). After applying RVC, the RC generally increased to 0.37 for the 20 mm sphere and reached 0.96 for the 40 mm sphere. After applying IY, the RC reached 0.75 for the 20 mm sphere and 1.04 for the 40 mm sphere. Table 3 presents the CoV for images at different noise levels. IY method was more robust to noise whereas RVC amplified image noise.

**Table 3:** The CoV of background at different noise levels.

Noise level	1	2	3	4	5
Non-PVC	0.07	0.13	0.25	0.28	0.37
RVC	0.13	0.29	0.45	0.58	0.85
IY	0.07	0.13	0.25	0.28	0.37

**Fig. 4:**
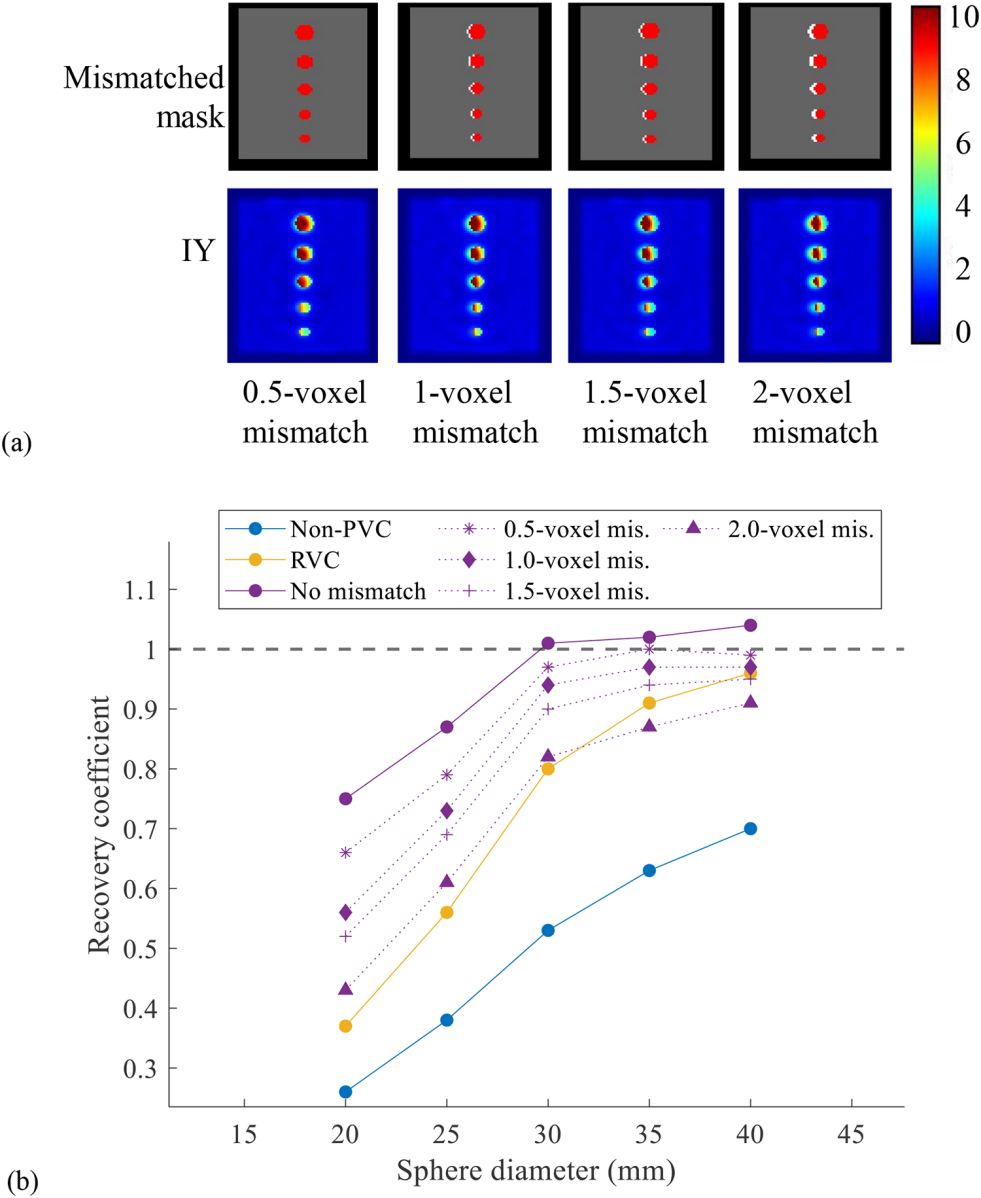
(**a**) The IY processed images using different mismatched masks. The red spheres indicate the mismatched mask while the white spheres are the standard mask. (**b**) The RCs of non-PVC, RVC and IY results using matched and mismatched masks.

Figure 4a shows the IY results at noise level 1 where sphere masks were mismatched with the SPECT images. The RCs of IY with matched and mismatched masks are shown in Fig. 4b. The activity error for all spheres with 1.0–2.0 voxels mismatch in IY mask was increased, with larger mismatches resulting in more errors. Additionally, IY results with mismatched masks were better than those of RVC for spheres with diameter ≤ 30 mm or when mismatch ≤ 1.5 voxels.

### XCAT phantom

**Fig. 5:**
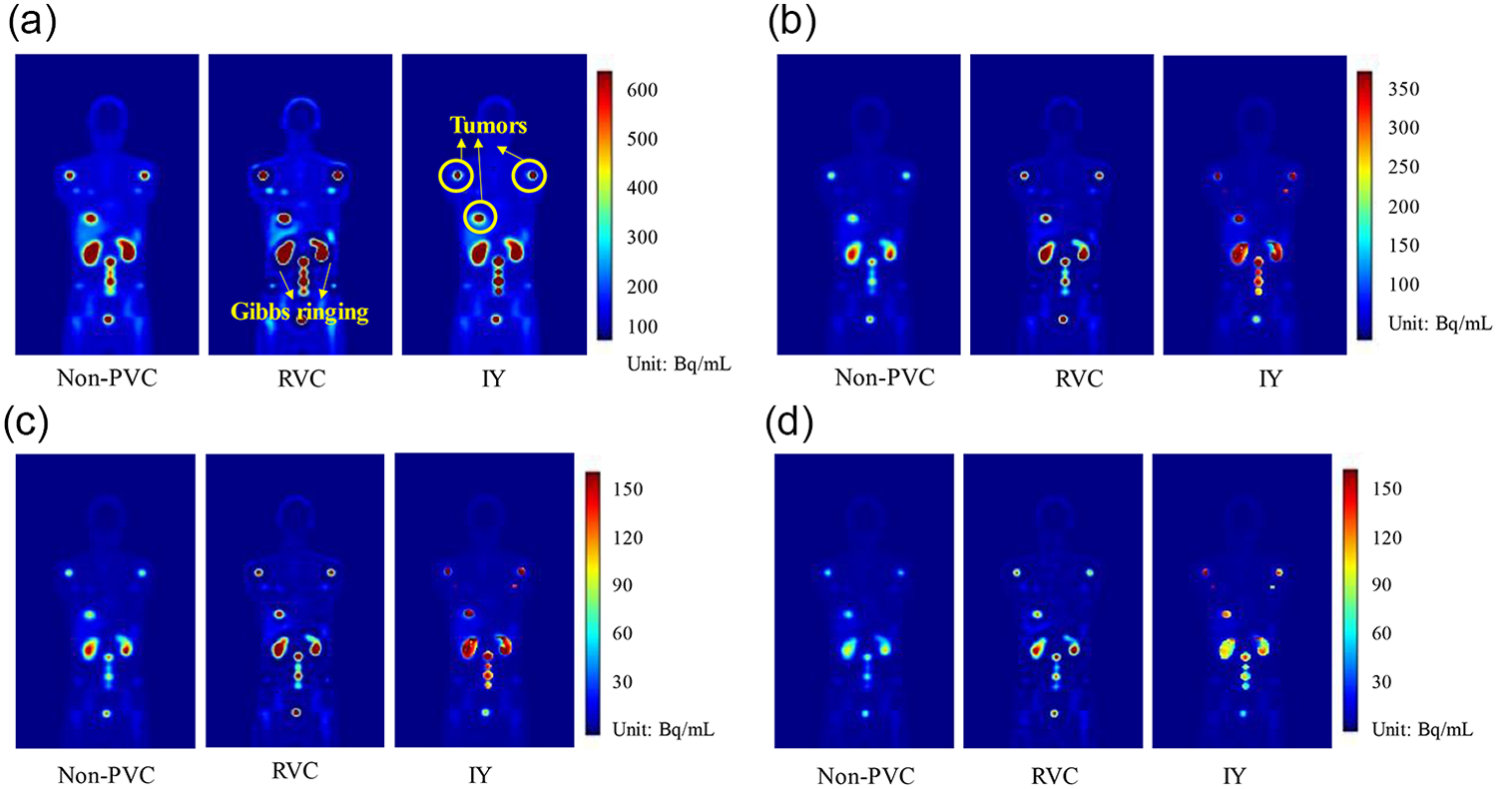
(**a**) Sample non-PVC reconstructed images and PVC results for a selected XCAT phantom at (**a**) 2, (**b**) 20, (**c**) 40, and (**d**) 200 h imaging time points.

The non-PVC, RVC and IY images for one sample XCAT phantom at four imaging time points are shown in Fig. 5. RVC method improved the activity estimation in the high uptake region but caused serious Gibbs ringing artifacts at the boundary between the high and low uptake regions, which were not observed in IY images. IY improved the activity estimation within the segmented kidney and tumor VOIs. Table 4 summarizes MAE ± standard deviation (SD) of kidneys and tumors, with and without PVC. The MAE ± SD in non-PVC of kidneys and tumors were 23.5%±4.1% and 56.9%±16.4%, respectively. RC-PVC, RVC and IY methods reduced the MAE ± SD of kidneys to 23.3%±3.9%, 15.7%±2.3% and 17.8%±2.5%, respectively. For tumors, RC-PVC, RVC and IY methods reduced the MAE ± SD to 25.4%±13.7% 33.9 ± 22.6% and 18.9%±23.6%, respectively. The mean activity differences of kidneys and tumors were 43.5%±4.1% and 76.6%±22.6% between non-PVC and RVC results, 54.3%±7.3% and 136.7%±53.3% between non-PVC and IY, 0.3%±0.3% and 103.9%±78.9% between non-PVC and RC-PVC, respectively.

**Table 4:** The MAE ± SD [Activity difference*] of kidneys and tumors for PVC and non-PVC results in 10 XCAT phantoms.

	Non-PVC	RC-PVC	RVC	IY
Kidney (*n* = 80)	23.5%±4.1%	23.3%±3.9% [0.3%±0.3%]	15.7%±2.3% [43.5%±4.1%]	17.8%±2.5% [54.3%±7.3%]
Tumor (*n* = 560)	56.9%±16.4%	25.4%±13.7% [103.9%±78.9%]	33.9%±22.6% [76.6%±22.6%]	18.9%±23.6% [136.7%±53.3%]

*Activity difference (%): the mean activity difference between PVC and non-PVC results

### Clinical data evaluation

**Fig. 6:**
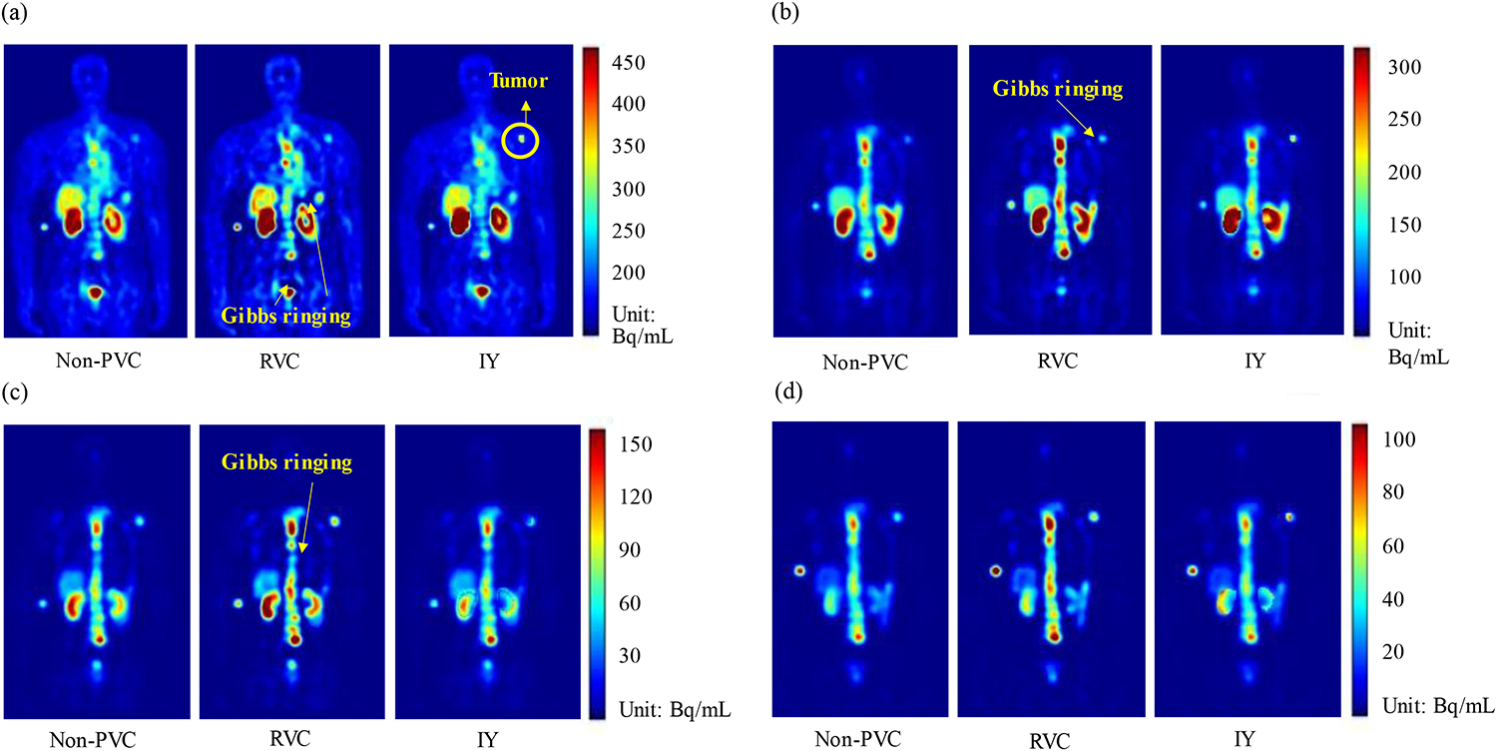
Sample clinical SPECT images, and corresponding RVC and IY results at (**a**) 2, (**b**) 20, (**c**) 40, and (**d**) 200 h imaging time points for a patient.

Figure 6 presents sample clinical ^177^Lu PSMA SPECT data and corresponding PVC results at four imaging time points of a sample patient. RVC method improved the activity estimation in the high uptake region but caused Gibbs ringing artifacts similarly as in XCAT phantoms. The activity in segmented kidney and tumor masks were enhanced on IY images. Table 5 summarizes the mean kidney and tumor activity differences among 10 patients between non-PVC and PVC results. The mean activity differences of kidneys and tumors, respectively, were 40.6%±9.2% and 63.5%±25.2% between non-PVC and RVC results, 51.3%±7.4% and 106.5%±71.2% between non-PVC and IY, 1.9%±1.0% and 82.3%±33.5% between non-PVC and RC-PVC.

**Table 5:** Mean activity difference for kidneys and tumors between non-PVC and PVC results on 10 clinical patients.

	RC-PVC	RVC	IY
Kidneys (*n* = 80)	1.9%±1.0%	40.6%±9.2%	51.3%±7.4%
Tumors (*n* = 40)	82.3%±33.5%	63.5%±25.2%	106.5%±71.2%

## Discussions

The influence of PVE varies depending on the size of the object. Generally, larger objects tend to be less affected by PVE. In the geometrical phantom study, the smaller spheres are more affected by PVE (RC of 0.26 for the smallest sphere to 0.70 for the largest sphere), consistent with previous reports [8, 24, 25]. The commonly used PVC methods (RVC and IY) enhance the activity estimation in general, and IY exhibits better activity recovery ability as compared to RVC, especially for smaller spheres (diameter < 30 mm). Furthermore, the XCAT phantom simulation study reveals that the MAE ± SD of kidneys and tumors are 23.5%±4.1% and 56.9%±16.4% for non-PVC. The use of RVC and IY reduces MAE ± SD of kidneys similarly (15.7%±2.3% vs. 17.8%±2.5%), probably due to the relatively large volume of kidneys. RC-PVC reduces the MAE ± SD of kidneys to 23.3%±3.9%. For tumors, RVC decreases the MAE ± SD to 33.9%±22.6%, while the MAE ± SD is 18.9%±23.6% for IY, consistent with the smaller sphere results.

The post-reconstruction filter is frequently applied to the clinical data to meet the visual preference of clinicians [26], while noise on the reconstructed images could be reduced with compromised quantitative accuracy. PVC results for unfiltered reconstructed images in the simulation study are shown in Supplementary Table S1 and Fig. S3-4, exhibiting less activity errors than those with post-filtering.

As an anatomically based method, IY often presents practical implementation challenges due to the absence of a ground truth VOI mask as well as the assumption of uniform activity distribution within the VOI [27]. For general practice, additional CT or MRI images are required to segment the VOI mask and then registered to SPECT images. Both RC-PVC and IY methods are effective in estimating tumor activity, as the volume or mask information is known. However, discrepancy in masks may impact the IY corrected activity. The distances between CT and SPECT caused by misregistration reach 5.7 ± 2.0 mm (range: 1.8–9.7 mm) in the neck, 6.8 ± 3.3 mm (range: 1.4–19.7 mm) in the abdomen [20], 8.6 ± 3.8 mm in the cardiac region [22], and range from 0.8 to 1.2 mm in the lower spine [23]. The spine is the common site of metastasis in mCRPC [2] and head-foot breathing motion in the left and right kidneys could be 7.2 ± 3.8 and 5.8 ± 4.1 mm [28], which can cause spatial blurring as well as discrepancies in CT and subsequent SPECT images. These discrepancies may result in mismatches when aligning VOIs obtained from CT to SPECT images [29]. In this study, the mismatched masks for IY (shifting distance from 2.54 to 10.16 mm) are sufficient to account for most of the motion amplitudes mentioned above. Although the RCs of small spheres (diameter < 30 mm) with mismatched masks in IY are lower than without mismatch (Fig. 4b), the RCs of spheres with mismatched masks still outperform those observed in non-PVC conditions. The RCs of small spheres (diameter < 30 mm) with mismatched masks in IY are better than those with RVC.

Notably, the originally proposed IY method (Eq. 5) implemented in PETPVC toolbox [19] does not preserve count and could lead to activity over estimations. Comparisons of IY results in the toolbox and our implementations with and without count preservation, are provided in the supplementary Fig. S5. Additionally, the uniform activity assumption in VOI implies that the variability within the VOI should be smaller than the variability between different regions [27]. Shcherbinin et al. [30] show that template-based correction methods based on uniform VOI masks perform best when the true activity is uniformly distributed. Recently, Leube et al. [31] introduce a deep learning (DL)-based PVC method for ^177^Lu SPECT/CT imaging, leveraging a training model based on 10,000 simulated datasets with random activity distributions. The DL-PVC demonstrates comparable activity recovery to IY methods without the need of anatomical information as input. Salvadori et al. [11] evaluate multiple anatomy‑based PVC methods from PETPVC toolbox [19] based on 3D printed kidneys attached in an International Electrotechnical Commission (IEC) phantom filled with ^177^Lu. Their results show that the kidney activity error ranges from 18 to 35% for non-PVC and is reduced to < 6% after PVCs. In our work, the MAE ± SD of kidneys is 23.5%±4.1% for non-PVC, consistent with their results. Our IY errors for kidneys are higher (MAE 17.8%±2.5%), which may be caused by the PSF mismatch in this study, as kidneys are not located at the center of FOV as compared to their study. Although RC-PVC is typically applied solely based on the selected VOIs, the position of the VOIs also influences the PVC performance [32]. PVC using spatially-variant PSF is currently under investigation in our laboratory and is beyond the scope of this study. Our paper provides additional insights concerning PVE/PVC on tumor quantitation, showing the effectiveness of RVC without inputting anatomical information, as well as the robustness of IY for noisy images and spatial mismatch.

On the other hand, the deconvolution-based RVC method without using anatomical information demonstrates the ability of voxel-level correction and insensitivity to discrepancies in masks. The need for voxel-level correction has been recognized in voxel-based targeted radionuclide therapy dosimetry [33]. However, the performance of RVC is greatly hampered by issues such as noise amplification and the Gibbs ringing artifacts. Even in images with low noise levels, RVC largely amplifies noise (Table 3). To address this problem, noise reduction models have been integrated into the deconvolution process, such as parallel level set regularization [34], highly constrained back-projection denoising [35], and normal inverse Gaussian distribution model [36]. However, these methods are still unable to fully correct the PVE. Consequently, implementing a non-negativity constraint could possibly aid the conventional RVC and is currently under investigation in our laboratory.

There are certain limitations in this study. First, our simulation study is based on the use of a circular detector orbit. Modelling of more clinically realistic body contour-based orbits is warranted to enhance the relevance and applicability of our findings. Second, our RC-PVC assumes a sphere-to-background ratio (10:1) equivalent to that of the geometrical phantom data. This assumption does not fully account for various tumor-to-background ratios in clinical data, potentially affecting the accuracy of RC-PVC in diverse patient population. Future works should consider refining the RC-PVC to account for the background spill-in [37]. Third, this study lacks a gold standard for activity measurement in the clinical study. However, despite these restraints, the XCAT phantom study and clinical data showed a similar trend in the kidney and tumor activity differences between non-PVC and PVC results (Tables 4 and 5), inferring the effectiveness of PVC in our study.

## Conclusions

We systematically analyze the influence of PVE and PVC on ^177^Lu-PSMA SPECT image quantification based on digital phantoms and clinical data. All PVC methods improve quantification accuracy effectively. RVC shows that PVEs still remain in small objects, with pronounced Gibbs ringing artifacts and noise amplification. Both RC-PVC and IY can improve the quantification in small structures. However, RC-PVC cannot produce corrected images and IY is subject to availability and mismatch of VOI masks.

## Electronic supplementary material

Below is the link to the electronic supplementary material.

Supplementary Material 1

## Data Availability

Authors will share data upon request to the corresponding author.

## Abbreviations

AC: Attenuation correction
CoV: Coefficient of variation
DL: Deep learning
FWHM: Full-width-at-half-maximum
GCDR: Geometrical collimator detector response
IEC: International Electrotechnical Commission
IY: Iterative Yang
MAE: Mean absolute error
mCRPC: Metastatic castration-resistant prostate cancer
MEGP: Medium energy general purpose
MRI: Magnetic resonance imaging
OS-EM: Ordered subset expectation maximization
PSMA: Prostate-specific membrane antigen
PSA: Prostate-specific antigen
PSF: Point spread function
PVE: Partial volume effect
PVC: Partial volume correction
RC: Recovery coefficient
ROR: Radius-of-rotation
RVC: Reblurred Van Cittert
SD: Standard deviation
TEW-SC: Triple energy window scatter correction
VOI: Volume-of-interest
