# Supplementary material for: Partial volume correction for Lu-177-PSMA SPECT
Source: EJNMMI Phys. 2024 Nov 12;11:93. doi: 10.1186/s40658-024-00697-1 (PMC11555037; doi:10.1186/s40658-024-00697-1)
Supplement: Supplementary file 1 — Supplementary Material 1 [file 40658_2024_697_MOESM1_ESM.docx]

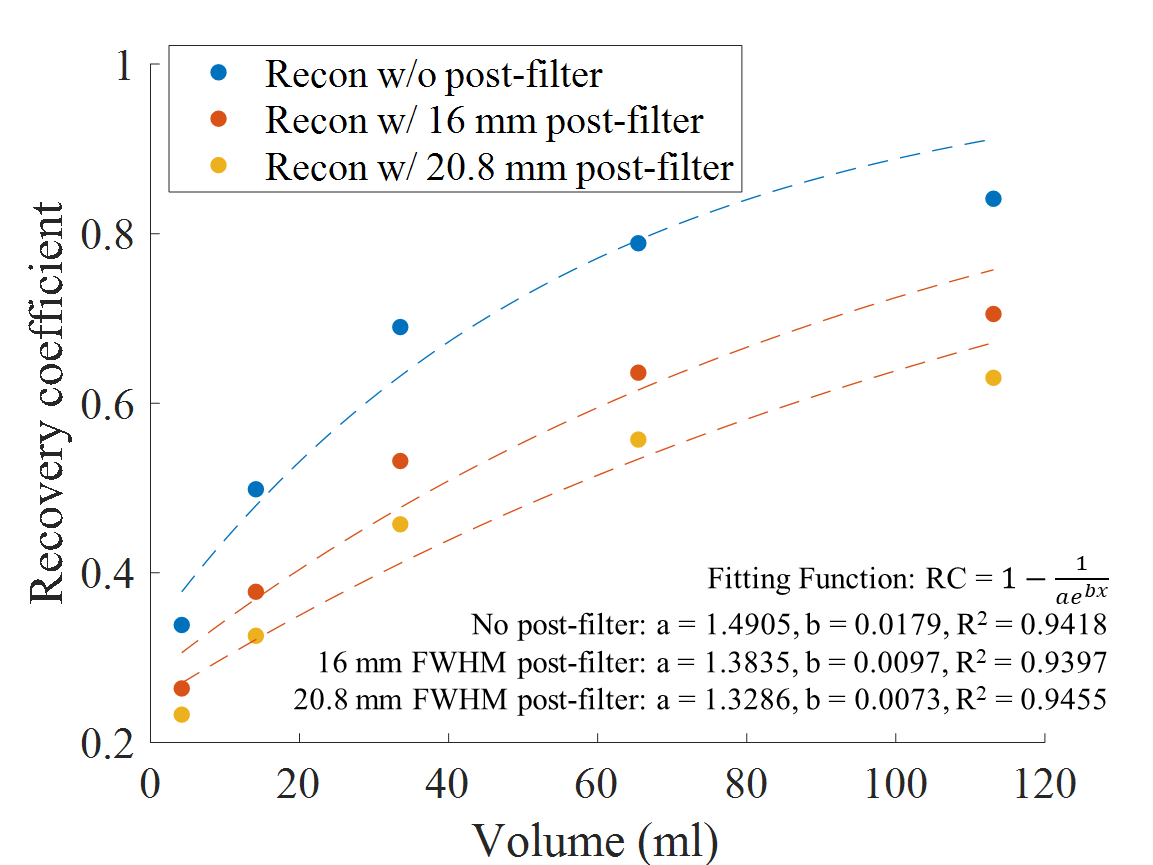


Figure S1. The fitted RC-curve for reconstruction with different post-filters. Dash line indicates the fitted curve, x represents the volume in ml.

(a)
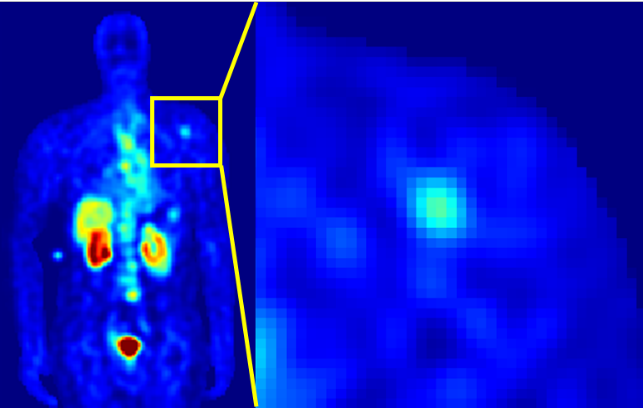


(b)
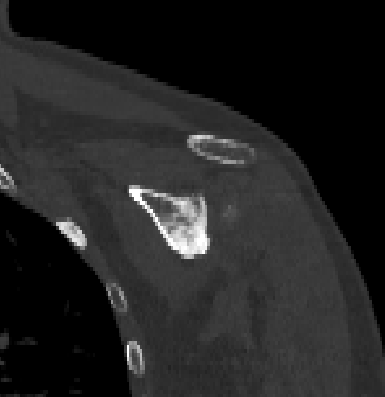
 (c)
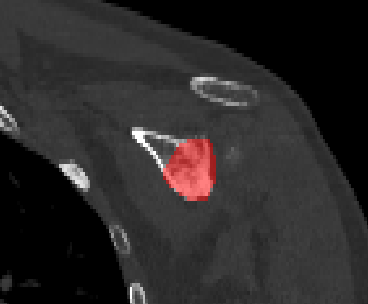


Figure S2. Sample (a) SPECT, (b) CT images and (c) the segmented tumor masked in red.

Figure S3. The non-PVC without post-filtering and corresponding PVC results of the geometrical phantom.


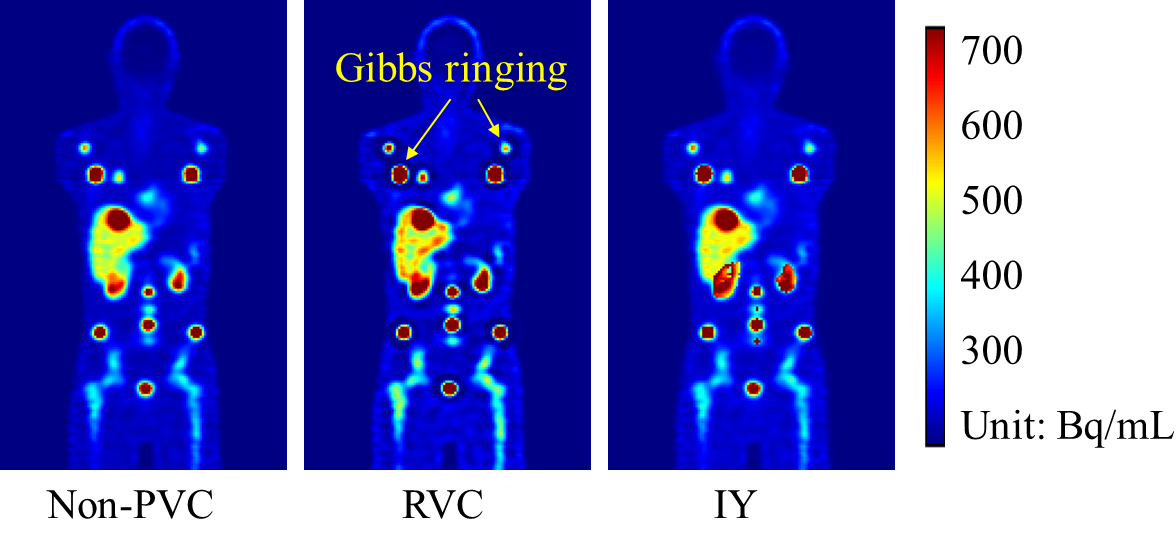


Figure S4. Sample non-PVC reconstructed images without post-filtering and corresponding PVC results for a selected XCAT phantom at 2 h imaging time point.

Figure S5. The comparison of RC for each sphere for non-PVC and PVC results in the PETPVC toolbox (11) and our implementation (BIG). The non-PVC is reconstructed with post-filter. The FWHM of 22.6 mm and matched VOI masks were used in the PVC methods from the PETPVC toolbox.

Table S1. The MAE±SD of kidneys and tumors for reconstructions without post-filtering and corresponding PVC results in 10 XCAT phantoms.

|  | Recon w/ GCDR, w/o filter | RC-curve | RVC | IY |
| --- | --- | --- | --- | --- |
| Kidney (n=80) | 9.7%±1.5% | 9.6%±1.5% | 8.2%±2.6% | 7.7%±1.9 % |
| Tumor (n=560) | 36.4%±19.6% | 17.7%±15.2% | 21.4%±23.0% | 16.3%±22.5% |
